# Resilience through adaptation

**DOI:** 10.1371/journal.pone.0171833

**Published:** 2017-02-14

**Authors:** Guus A. ten Broeke, George A. K. van Voorn, Arend Ligtenberg, Jaap Molenaar

**Affiliations:** 1 Biometris, Wageningen University & Research, Wageningen, Netherlands; 2 Laboratory for Geo-information Science and Remote Sensing, Wageningen University & Research, Wageningen, Netherlands; University of California, UNITED STATES

## Abstract

Adaptation of agents through learning or evolution is an important component of the resilience of Complex Adaptive Systems (CAS). Without adaptation, the flexibility of such systems to cope with outside pressures would be much lower. To study the capabilities of CAS to adapt, social simulations with agent-based models (ABMs) provide a helpful tool. However, the value of ABMs for studying adaptation depends on the availability of methodologies for sensitivity analysis that can quantify resilience and adaptation in ABMs. In this paper we propose a sensitivity analysis methodology that is based on comparing time-dependent probability density functions of output of ABMs with and without agent adaptation. The differences between the probability density functions are quantified by the so-called earth-mover’s distance. We use this sensitivity analysis methodology to quantify the probability of occurrence of critical transitions and other long-term effects of agent adaptation. To test the potential of this new approach, it is used to analyse the resilience of an ABM of adaptive agents competing for a common-pool resource. Adaptation is shown to contribute positively to the resilience of this ABM. If adaptation proceeds sufficiently fast, it may delay or avert the collapse of this system.

## Introduction

Many social-ecological systems, which provide important ecosystem services, are under increasing pressure from human activities and environmental changes [1, 2]. To predict how these systems will respond to pressures, we need to describe their Complex Adaptive System (CAS) characteristics. CAS are systems with many autonomous agents that interact with each other and with their environment [3]. The system-level behaviour of CAS ‘emerges’ from lower-level interactions and cannot a priori be predicted from the properties of its agents. To properly manage CAS that are under pressure, it is important to understand which properties affect resilience, i.e., the capacity of the system to cope with pressures while maintaining its identity and avoiding drastic changes [4]. It has been shown that some CAS show an initial resilience against pressure, until a tipping point is reached where the system undergoes a drastic transition to an entirely different system state [5]. In order to predict the occurrence of such transitions, we need to understand the origin and extent of the resilience of CAS [6].

**Fig 1:**
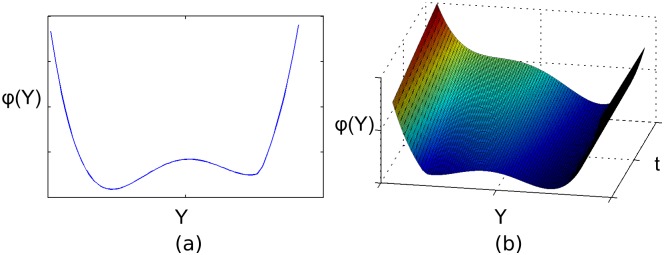
Hypothetical potential landscape *ϕ*(*Y*) as function of a state variable *Y*. (a): The landscape is static in time. The black ball represents the current system state. The two valleys correspond to two separate stability domains. (b): The landscape changes over time. At *t* = 0 the landscape is identical to (a). Over time, the leftmost stability domain becomes less resilient and eventually disappears.

Real-world CAS constantly experience the influence of small disturbances, changing conditions, and random events. This means that CAS are never in a static equilibrium situation and their state is continuously changing [4]. Most of these changes are small, and do not affect the organisation of the system fundamentally. Such small changes are commonly described as movements within a ‘domain of attraction’. Within a domain of attraction, the system maintains the same qualitative structure and organisation. Often these domains of attraction are illustrated metaphorically as valleys in a potential landscape that describes the state of the system. For example, Fig 1a shows a hypothetical potential landscape with two domains of attraction. The ball represents the current state of the system, and is naturally attracted towards the bottom of the domain of attraction. Pressures and shocks of limited strength and duration continuously shake the system within the boundaries of the current domain of attraction, but rarely push the system past those boundaries. Pressures and shocks that are strong and/or frequent, in contrast, are more likely to push the system outside its domain of attraction. Such a tipping point leads to a qualitatively different kind of system state, and may have drastic consequences for the development of the system. The resilience of the system against the occurrence of tipping points is, of course, determined by the shape of the current stability domain. If this stability domain is wide and deep, external pressures are unlikely to cause a tipping point, and the system is said to be resilient.

In many studies, resilience is assessed by modelling the system with an ordinary differential equation model and using bifurcation analysis to detect the boundaries of the domains of attraction (e.g., [7]). In this approach, the stability landscape is assumed to be static over the course of the simulation. In the context of CAS, however, this assumption is an oversimplification [4]. Changing conditions may affect the width, depth, and position of the domains of attraction. This is illustrated in Fig 1b, in which the potential landscape is initially identical to Fig 1a, but over time the leftmost domain of attraction gradually becomes less resilient and eventually disappears. As a result, the system will undergo a transition into the remaining stability domain. Adaptation is considered to be a key factor for understanding how the resilience of CAS changes over time [1, 6]. Adaptation refers to the capacity of agents to affect the resilience of the system through adjustment of their behavioural rules in response to perceived or expected changes in the system. This adaptation may refer to Darwinian natural selection, but also to agents trying to learn to cope with their environment, for instance through trial-and-error, or through imitation of other agents (e.g., [8–10]). Adaptation ensures that agents can change the resilience of the system by adjusting the boundaries of the domain of attraction, for instance by becoming better adapted to conditions that put pressure on the system. Thus, in terms of the potential landscape in Fig 1b, adaptation might prevent the loss of resilience of the leftmost stability domain, by deepening this domain.

The effects of agent adaptation in CAS are often modelled using agent-based models (ABMs), which explicitly model interactions between agents in an environment [3]. In [10] a distinction is made between adaptive and non-adaptive ABMs. Adaptive ABMs are defined as ABMs ‘in which the interacting, autonomous agents change their behaviors during the simulation, as agents learn, encounter novel situations, or as populations adjust their composition to include larger proportions of agents who have successfully adapted’ [10]. In contrast, in non-adaptive ABMs the behavioural rules of agents remain constant throughout the simulation. In this paper we will follow this definition of adaptive ABMs. Nowadays, the usefulness of ABMs is still limited by a lack of available methodologies for analysing and drawing robust conclusions from the model behaviour [10–15]. Standard methodologies of model analysis often yield insufficient information to uncover key relations between the model inputs and its outputs [16, 17]. Furthermore, there is currently no standardised methodology to determine the boundaries of domains of attraction in ABMs [18]. Agent adaptation further complicates this issue, because it is necessary to take into account that agents have the capacity to change the shape of the stability domain [19–21]. Besides adaptation, the presence of stochasticity is another factor that may complicate the analysis of many ABMs. Many ABMs contain stochasticity, and this introduces intrinsic variation in the model output.

In this paper we propose a methodology to analyse adaptation and its effects on resilience using ABMs. We apply the methodology to a previously published ABM [16] of consumers competing for a common-pool resource. The methodology is based on a comparison between an ABM with adaptation and a version where adaptation has been disabled. For both versions we account for stochasticity of the model output by estimating probability density functions (pdfs) that assign probabilities to each possible output value. Since adaptation is a process that takes place over time, we estimate both pdfs as a function of time. If the boundaries of the current domain of attraction of the ABM are crossed, then the output pdf shows a transition to a different system state. We use methods of sensitivity analysis to locate such boundaries. For the ABM without adaptation these boundaries are fixed, but for the ABM with adaptation the boundaries may shift as agents become better adapted. The difference between the pdfs of both ABMs is used to measure the effects of adaptation on the resilience of the system. We quantify this difference using the so-called earth-mover’s distance.

## Materials and methods

### Stochastic output

Since most ABMs are stochastic, each single simulation run of an ABM yields its own output. We use a large number of replicate runs to estimate the range of possible output values. Most sensitivity analysis methods use these replicate runs only to estimate the mean and variance of the model output under various parameter settings (e.g., [22]). If the model output is normally distributed, then the mean and variance indeed fully describe its variation. For ABMs, however, both the underlying model behaviour and the corresponding shape of the output distribution are usually not known a priori. This shape contains information that is important for understanding the model behaviour. For example, if a number of model runs undergo a transition into another domain of attraction, this will lead to a bimodal output distribution.

**Fig 2:**
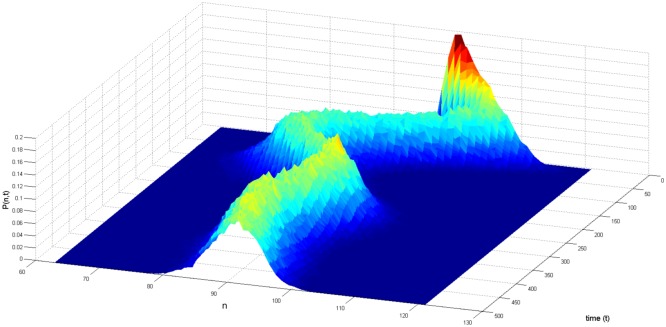
Time-dependent histogram. Time-dependent histogram that estimates the probability *P*(*n*, *t*) of obtaining some model output *n* at time *t*. The figure was generated using 10.000 replicate runs. In this example, the output seems to stabilise as a normal distribution around of mean value of *n* = 90.

In this paper, we use replicate runs to estimate the output distribution. These estimates will be visualised as histograms. Since the output distributions are time-dependent, they are estimated as functions of time. Plots of the time-dependent estimated pdfs visualise the change of the model output over time. For example, the pdf Fig 2 initially shows fluctuations over time, after which it converges to a domain of attraction. After this convergence it seems to stabilise as a normal distribution. Since adaptation is a process that takes place over time, its effects are contained in the time-dependent pdf. Critical transitions where the model is pushed into an alternative domain of attraction appear as sudden changes in the pdf.

### Statistical tests

To assess the characteristics of the estimated output pdfs, we use statistical tests for stationarity and ergodicity. Details of these tests are given in S1 Appendix. Stationarity tests use a number of replicate runs to verify whether an ABM becomes ‘stationary’, i.e. reaches a state in which its output pdf shows no long-term changes over time [23]. For example, in Fig 2 the model appears to converge to a stationary state, after some initial fluctuations. If the ABM becomes stationary, the corresponding pdf represents the long-term behaviour of the ABM and there is no adaptation over the considered time-span.

Ergodicity tests compare an output sample of a single model run, which is obtained by recording the model output every time-step, to output samples of a number of replicate runs measured at a set time [23, 24]. The model is considered to be ergodic if the null-hypothesis that the output samples are identical is not rejected. In other words, the stochastic variation between replicates is then equal to the stochastic variation over time of a single model run. A difference between the output samples indicates that the model output contains variation over time that cannot be attributed to stochasticity, and may indicate that adaptation has taken place over the considered time-scale. Since only a single model run is used to estimate the time-averaged pdf, ergodicity is a highly attractive property if we want to explore the model behaviour on very long time-scales. In the following, we will refer to histograms that are obtained by recording a single model run over successive times-steps as *time-averaged* histograms. We refer to histograms that are recorded over replicate runs as function of time as *time-dependent* histograms.

### Sensitivity measures

In order to assess how adaptation influences the model output, we need to measure the sensitivity of the output pdf to the presence of adaptation. We measure this sensitivity by comparing the estimated time-dependent pdfs of the output of model versions with adaptation *P*_*a*_(*n*, *t*) and without adaptation *P*_*b*_(*n*, *t*). Here *n* denotes the number of agents, which is used throughout this paper as the central model output. To quantify the comparison we use the earth-mover’s distance *d*_*e*_ [25].

For an intuitive interpretation of the earth mover’s distance, consider two pdfs *P*_*a*_(*n*, *t*) and *P*_*b*_(*n*, *t*) as amounts of mass that are spread over a distance specified by the model output *n*. The earth-mover’s distance *d*_*e*_(*P*_*a*_(*n*, *t*), *P*_*b*_(*n*, *t*)) is then the work required to transform *P*_*a*_(*n*, *t*) into *P*_*b*_(*n*, *t*). Several properties follow from this interpretation. Firstly, for all *P*_*a*_(*n*, *t*) and *P*_*b*_(*n*, *t*), *d*_*e*_(*P*_*a*_, *P*_*b*_) ≥ 0, and *d*_*e*_(*P*_*a*_, *P*_*b*_) = 0 implies that *P*_*a*_ = *P*_*b*_. Any positive value of *d*_*e*_(*P*_*a*_, *P*_*b*_) thus indicates a difference between the pdfs. Small values indicate that the pdfs are quite similar, and large values indicate strong differences between the pdfs. We denote by

$$Q\left(t\right)=d_{e}\left(P_{a}\left(n,t\right),P_{b}\left(n,t\right)\right)$$ (1)

the effects of adaptation at time *t*. Note that all the used symbols are described in Table 1. *Q*(*t*) measures the effect of a parameter change, namely the enabling or disabling of adaptation. Other sensitivity measures based on a comparison between output pdfs have been previously suggested (e.g. [26–28]). All of these measures are based on the (lack of) overlap between those pdfs. For our purposes, these measures are not suitable, because we want to measure the effects of adaptation even when there is little or no overlap between the pdfs. The earth-mover’s distance allows us to do this, because an increase in the distance between pdfs will result in an increase of the earth-mover’s distance, even when the pdfs do not overlap.

**Table 1:** Description of used symbols.

Symbol	Description	Units
*D*	Diffusion coefficient	km^2^day^−1^
*d*_*e*_	Earth mover’s distance	-
*d*_*J*_	Jensen-Shannon distance between histograms	-
*d*_*u*_	Euclidian distance between histograms	-
*d*_(_*j*, *k*)	Distance between bins of histograms (S2 Appendix)	-
*E*_*h*_	Energy cost for an agent to harvest	J
*E*_*m*_	Energy cost for an agent to move	J
*g*_(_*j*, *k*)	Flow between bins of histograms (S2 Appendix)	-
*H*_*t*_	Vector used in runs test (S1 Appendix)	-
*j*, *k*, *l*	Indices for output values	-
*N*	Number (observations, model runs,…)	#
*n*	Number of agents	#
*P*_*a*_(*n*, *t*)	Normalised histogram of ABM with adaptation	-
*P*_*b*_(*n*, *t*)	Normalised histogram of ABM without adaptation	-
*Q*_*i*_(*t*)	Adaptation measure for parameter i at time t	-
$Q_{i}^{\prime }\left(t\right)$	Rate of adaptation over time	t^−1^
*T*	Length of time-series	-
*t*	Time index	day
*V*	Variance	-
*W*	Statistic used in trend test (S1 Appendix)	-
*w*_*harvest*_	Agent’s harvest parameter	-
*w*_*move*_	Agent’s move parameter	-
*Y*	Model output variable	-
*z*	Inheritance parameter	-
*λ*	Extinction parameter	day^−1^
*μ*	Mean	-
*ρ*	Increase in pressure	-
*ϕ*	Potential	J

In addition to the effects of adaptation at a specific time, we are also interested in the rate of adaptation. We calculate the rate of change of *Q*(*t*) by computing the difference between time-steps,

$$Q^{\prime }\left(t\right)=d_{e}\left(P_{a}\left(t\right),P_{b}\left(t\right)\right)-d_{e}\left(P_{a}\left(t-\Delta t\right),P_{b}\left(t-\Delta t\right)\right),$$ (2)

with Δ*t* small compared to the time-scale of the process. This measure for the rate of adaptation helps to identify periods in time where adaptation proceeds relatively fast, or where there is little adaptation. For example, adaptation may be influential on short simulation times, but have little effect on longer simulation times, or the other way around.

### Computation of the earth mover’s distance

Computing the earth-mover’s distance between a pair of distributions amounts to finding the minimal ‘work’ needed to change one distribution into the other. We write *g*(*j*, *k*) for the matrix element that contains the flow between output values *j* and *k*, *d*(*j*, *k*) for the distance between the output values, and we consider *g*(*j*, *k*)*d*(*j*, *k*) as the work required to transport *g*(*j*, *k*) from *j* to *k*. There are many possible choices for the matrix *g* to transform one distribution into the other. To compute the earth-mover’s distance, we minimise *g*(*j*, *k*)*d*(*j*, *k*) over these possible choices [29],

$$d_{e}=\text{min}\left(\sum_{\left(j,k\right)}g\left(j,k\right)d\left(j,k\right)\right)$$ (3)

with the constraints

$$\sum_{k}g\left(j,k\right)=P_{a}\left(j\right)\;\forall j$$ (4)

$$\sum_{j}g\left(j,k\right)=P_{b}\left(k\right)\;\forall k$$ (5)

g(j,k)≥0∀j,k (6)

with *d*(*j*, *k*) the distance between *j* and *k*, and the indices *j* and *k* running over their domains. Eqs 4 and 5 ensure that the flow is such that the distribution *P*_*a*_(*j*) is transformed into *P*_*b*_(*k*). Eq 6 ensures that mass is moved from *P*_*a*_(*j*) to *P*_*b*_(*k*), and not the other way around. Eq 3 ensures that the flow is chosen such to minimise the required ‘work’ *g*(*j*, *k*)*d*(*j*, *k*). For two pdfs of a single output variable, this minimisation is accomplished by going through all consecutive pairs of output values, and keeping track of the amount of mass that needs to be transported. S3 Appendix contains a sample pseudo-code for this computation. For the computation of *d*_*e*_ for continuous, or multi-dimensional distributions, we refer to [30]. Packages to compute *d*_*e*_ are available for various statistical software and programming languages.

### Method to measure adaptation

**Fig 3:**
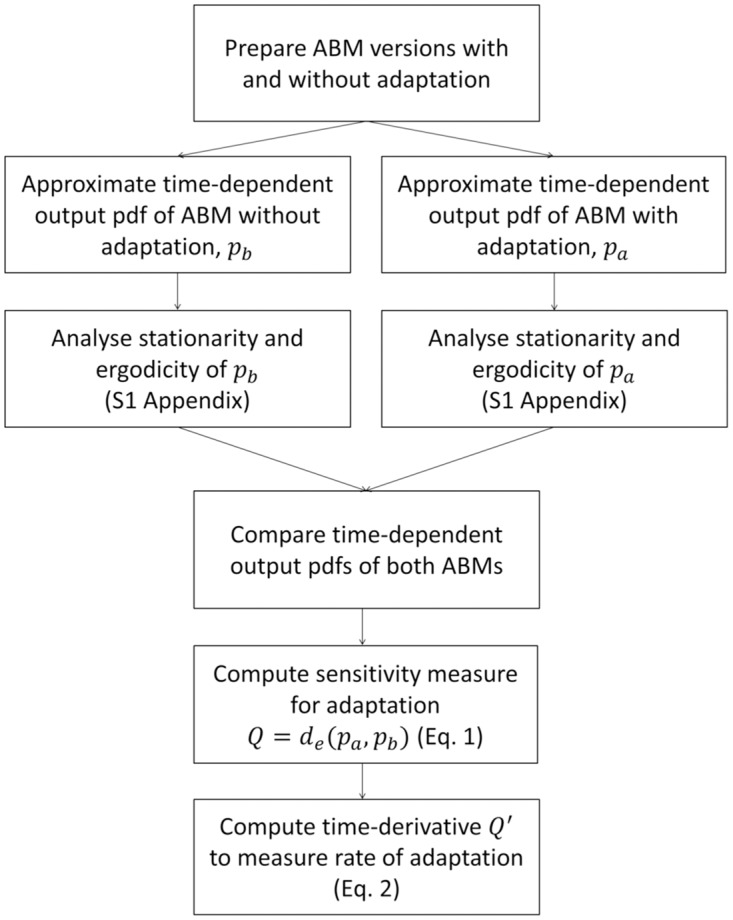
Flow chart of the proposed method to measure adaptation. The method is based on a comparison between versions of an ABM with and without adaptation. See the main text for further explanation.

In Fig 3 we present the scheme of our method to measure adaptation of ABMs. The first step is to prepare an ABM with adaptation, and a version of the same ABM in which this adaptation is disabled. Agent adaptation in our test-case consists of the selection of agent characteristics for replication or modification [6]. Disabling this selection ensures that there is no adaptation and that the distribution of agent properties does not change over time. In the ABM with adaptation, the agent properties may change over time, for example through natural selection, or through individual agents learning from past experience or imitation of other agents [3, 10]. To measure the effects of such adaptations, we compare the output of the ABM with adaptation to that of the ABM without adaptation. The rest of the method is composed of steps for analysing the model output and comparing the estimated output pdfs using Eqs 1 and 2.

Based on a large number of replicate runs, we generate histograms of the output to estimate the time-dependent output pdfs of the ABM without adaptation *P*_*b*_ and of the adaptive ABM *P*_*a*_. To measure adaptation, we need to examine how the pdfs change with time. Thus, for each ABM we estimate the pdf at multiple points in time. Based on these data, we use stationarity tests to verify whether the ABMs reach an equilibrium, or continue to change over the considered time-scale. Furthermore, for each ABM we also perform a single model run with a very long simulation time to test the ergodicity. If the stationarity test reveals that the ABM reaches a stationary state, then the time after which this state is reached is used as a starting point for the ergodicity test, but the ergodicity test explores longer simulation times than the stationarity test. If the model is ergodic, then we may conclude that no adaptation takes place.

Once the pdfs of the ABMs with adaptation and without adaptation have been analysed, we proceed by comparing them to quantify the effects of adaptation. The difference between the pdfs is quantified using *Q* (Eq 1). In addition to measuring the effects of adaptation over a specified time-span, we also measure the rate of adaptation using *Q*′ (Eq 2). This rate of adaptation is especially relevant in the context of resilience. For the resilience of the system to be influenced by adaptation, the rate of adaptation must be sufficiently fast to respond to pressures. For example, if a pressure to the system increases very fast, then adaptation must also be able to occur fast in order to affect the consequences of the pressure.

### Model description

**Fig 4:**
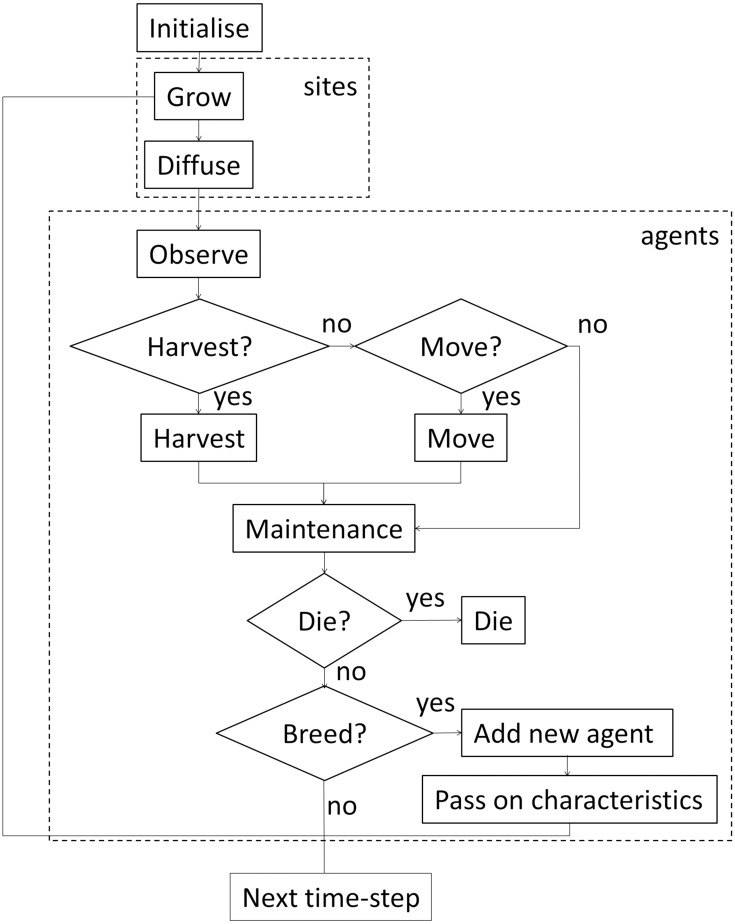
Model flow chart. The dashed boxes indicate loops over all sites or all agents. For detailed explanation we refer to [16].

**Fig 5:**
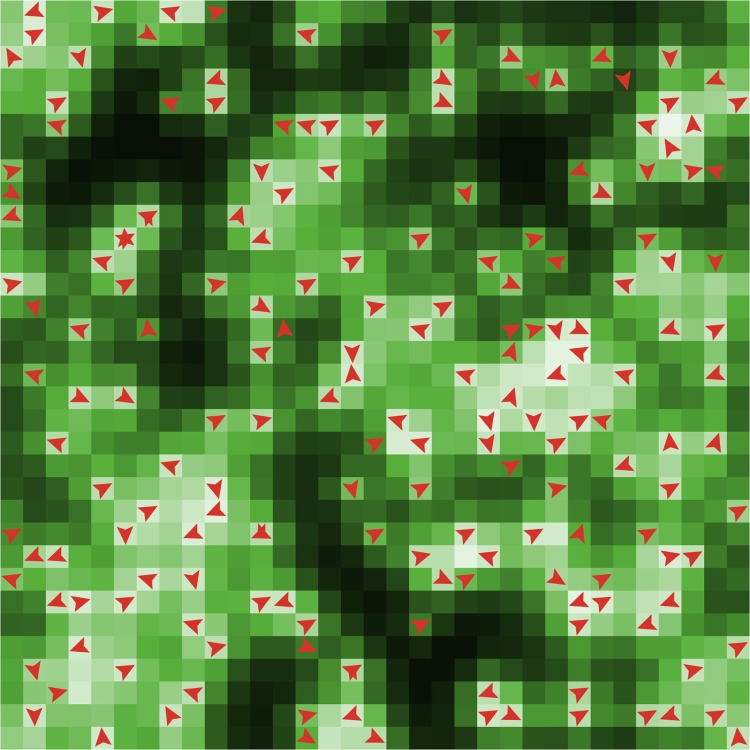
Snapshot of a typical simulation run of the ABM test-case. The ABM is composed of a square grid of sites. Dark colours indicate sites with high resource densities, while light colours indicate low densities. The red arrows show the current locations of agents.

We consider a previously published test-case, in which adaptation emerges through a process of natural selection. An overview of all model parameters and their default values is given in S1 Table. For a full model description we refer to [16]. Here we focus mainly on the mechanism for adaptation. Fig 4 presents a flow chart of the model. The test-case is a resource-agent system, in which the agents compete for a renewable common-pool resource in a spatial environment. The spatial environment is composed of a grid of square sites, on which resource grows and diffuses, and on which agents live (Fig 5). Every time-step, each agent estimates the amounts of resource and observes the number of agents on its present location and the four neighbouring sites. Based on this information, it decides whether to harvest on its present site, to move to a neighbouring site, or to stay inactive. These decisions are stochastic, but the probabilities are influenced by the agent’s own (decreasing) internal energy state (‘hunger’) as well as the state of the local surroundings, including the presence of resources (‘food availability’) and other agents (‘crowding’). Harvesting or moving costs energy, in addition to the energy consumed by basic maintenance every time-step. There are thus trade-offs between harvesting, moving, and remaining inactive, which form the basis of the agent’s decision-making process. At the end of each time-step, all agents have a probability of dying and of reproducing, based on their internal energy state. Low values for the internal energy increase the probability of dying, whereas high values increase the probability of reproducing.

Each agent has two agent parameters that affect its decisions to harvest or move: *w*_*harvest*_ and *w*_*move*_. Besides these parameters, the probabilities of harvesting or moving also depend on the weighted average of a few factors, including the agent’s internal energy, the resource on its site and the resource on its neighbouring sites. This average, multiplied by *w*_*harvest*_ or *w*_*move*_, is input to a function that determines the probability. If *w*_*harvest*_ = 0 the agent will choose to harvest with probability 1. Similarly, if *w*_*move*_ = 0 the agent will choose to move with probability 1. Larger parameter values decrease the probabilities. For the initial agent population, the values of these parameters are drawn from a uniform probability distribution. The values remain constant over the lifetime of an agent. Upon reproduction, an agent passes on its values of *w*_*harvest*_ and *w*_*move*_ to its offspring, with a small random deviation. The distribution of the parameter values across the population may thus change over time through a process of natural selection. Since the probability of reproducing is higher for agents with a high internal energy, agents that are successful at gathering energy are also more likely to reproduce and pass on their characteristics. Agents that are not successful at gathering energy, in contrast, are more likely to die without reproducing. Parameter values that increase an agent’s success at gathering energy are thus passed on more frequently, whereas parameter values that decrease its success tend to disappear from the population. Over time, the distribution across the population of the parameters values will thus move towards values that increase success at gathering energy. This change in the distribution represents the process of the population adapting to its environment.

To obtain a non-adaptive version of the ABM, we disable the inheritance of agent characteristics. In the non-adaptive version, when a new agent is added to the system, its values of *w*_*harvest*_ and *w*_*move*_ are chosen according to the same probability distribution that is used for the initial agent population. Thus, in this version there are no long-term changes in the distribution of *w*_*harvest*_ and *w*_*move*_ across the agent population.

## Results

### Non-adaptive ABM

In the following sections, we will consider the agent population size *n* as the central model output. Following the scheme in Fig 3, we estimate the output histogram of the ABM without adaptation in the default parameter setting. Detailed results of the statistical tests are given in S3 Appendix. Model runs in the default parameter setting show that the output initially oscillates, but stabilises around *t* = 1000. Between *t* = 1000 and *t* = 2000, the mean of the output across replicates is nearly constant on average, with short-term fluctuations. Due to these fluctuations, stationarity tests show that the output is not stationary. To test whether there are any long-term trends, we average the output over short time-windows and test whether the series of window means is stationary [31]. These tests indicate that between *t* = 1000 and *t* = 2000 the window means are stationary.

**Fig 6:**
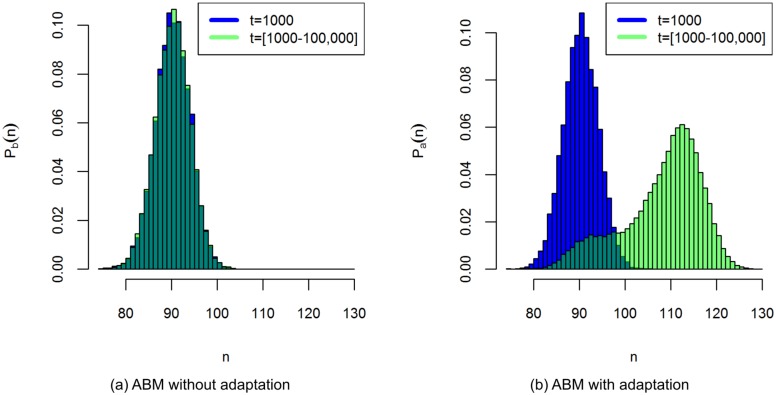
Time-averaged histograms of the number of agents (*n*). The blue histograms are measured over 10,000 replicate runs at *t* = 1000, and the green histograms over the time-steps of a single model run between *t* = 1000 and *t* = 100,000. All parameters are at their default values. Fig 6a indicates that the ABM without adaptation is ergodic, and Fig 6b indicates that the ABM with adaptation is not ergodic.

To test the ergodicity we compare the histogram *P*_*b*_ of the output over replicate runs at *t* = 1000, to the histogram of a single model run, ranging from *t* = 1000 to *t* = 100,000. The test confirms that the ABM without adaptation is ergodic, indicating that it shows no long-term change even on simulation times up to 100,000 time-steps (Fig 6a). The histogram over replicate runs at *t* = 1000 and the time-averaged histogram thus both estimate the stationary pdf of the ABM without adaptation, and describe the behaviour of this ABM on long time-scales.

### Adaptive ABM

The stationarity test for the adaptive ABM shows similar results as the ABM without adaptation. The model output has stabilised around *t* = 1000 and appears to be stationary between *t* = 1000 and *t* = 2000. The output histogram corresponding to the stationary state is approximately equal to the histogram of the ABM without adaptation.

Although the stationarity test does not reveal any effects of adaptation, it is possible that adaptation proceeds so slowly that the time period between *t* = 1000 and *t* = 2000 is too short to observe its effects. We use an ergodicity test to explore the behaviour of the ABM with adaptation on longer time-scales. The results reveal that the model output is indeed affected by adaptation on simulation times between *t* = 1000 and *t* = 100,000. The time-averaged histogram of a model run with a long simulation time differs significantly (S3 Appendix) from the histogram over replicate runs at *t* = 1000 (Fig 6b). Over time the agent population gradually adapts, causing a significant increase in the population size. So we observe that, while the system remains in the same stability domain on long time-scales, adaptation causes the shape of this stability domain to gradually change over time, whereas without adaptation the shape of the stability domain remains constant.

### Effects of adaptation

**Fig 7:**
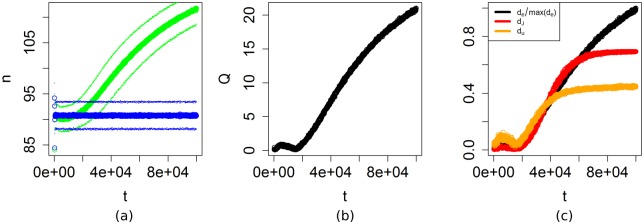
Effects of adaptation. (a): Means of the pdfs *P*_*a*_(*green*) and *P*_*b*_ (blue) at the default parameter settings. The thinner lines show the mean plus or minus one standard deviation. Both the mean and standard deviation were estimated based on 1000 replicate runs. (b): Plot of *Q* between the pdfs *P*_*a*_ and *P*_*b*_, as functions of time. The plot shows that on long time-scales the effects of adaptation increase. (c): Comparison between the earth-mover’s distance *d*_*e*_ (black), the Jensen-Shannon divergence *d*_*J*_ (red), and the Euclidian distance *d*_*E*_ (orange). To fit in the same graph, we plot *d*_*e*_ divided by its maximimum value. For large values of *t*, *d*_*J*_ and *d*_*u*_ become insensitive to changes in the pdfs when there is no longer overlap between the pdfs. In contrast, *d*_*e*_ continues to increase.

To estimate the effects of adaptation we compute *Q* between the estimated time-dependent pdfs *P*_*a*_ and *P*_*b*_. We use a total simulation time of 100,000 time-steps, and record the output every Δ*t* = 100 time-steps. Fig 7a shows that the distance between the pdfs increases on long time-scales. The corresponding values of *Q* (Fig 7b) show that the distance between the pdfs initially increases, but then decreases approximately between *t* = 5,000 and *t* = 15,000, after which it increases again. The initial increase of *Q* is caused by a small decrease in the output of the ABM with adaptation. After this initial decrease in the output, the output starts to increase, first moving towards the ABM without adaptation, and then becoming larger. The initial decrease of the number of agents in the adaptive ABM is caused by increased competition between the agents, which tend to harvest more often. On longer time-scales, the agents adapt to move less often, and wait at the same location to let the resource grow before harvesting. Since agents mostly stay in their location, there is a decreased competition between the agents, which leads to an increase in the number of agents on longer time-scales. This learning process continues until the simulation is stopped at *t* = 100,000 as is shown by the values of *Q* and *Q*′.

To demonstrate why we prefer the earth-mover’s distance over some other measures, we have reproduced Fig 7b using two other commonly used measures for the difference between pdfs, namely the Euclidian distance

$$d_{u}=\sqrt{\sum_{j}{\left|P_{a}\left(j\right)P_{b}\left(j\right)\right|}^{2}}$$ (7)

and the Jensen-Shannon divergence

$$d_{J}=\frac{1}{2}\sum_{j}\left[P_{a}\left(j\right)\text{ln}\left(\frac{2P_{a}\left(j\right)}{P_{a}\left(j\right)+P_{b}\left(j\right)}\right)+P_{b}\left(j\right)\text{ln}\left(\frac{2P_{b}\left(j\right)}{P_{a}\left(j\right)+P_{b}\left(j\right)}\right)\right].$$ (8)

Similar to *d*_*e*_ both measures initially measure an increasing difference between the pdfs (Fig 7c). For larger values of *t* this increase flattens off because the measures reach a maximum when there is no overlap between the pdfs. The earth mover’s distance *d*_*e*_, in contrast, continues to increase because the distance between the means of the pdfs is still increasing.

### Resilience

**Fig 8:**
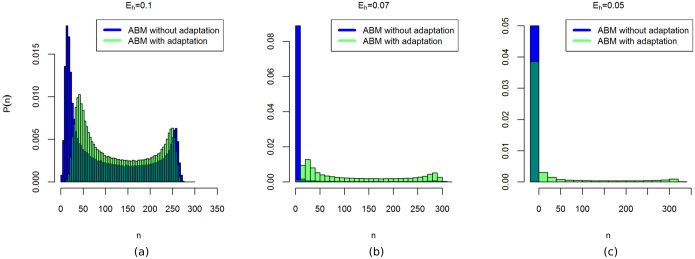
Time-averaged histograms of the number of agents *n*, for selected values of *E*_*h*_. The peaks at *n* = 0 correspond to the population going extinct.

As shown in [16], the model contains tipping points where the population collapses and goes extinct. For example, Fig 8 shows that a tipping point is crossed by decreasing the harvest parameter *E*_*h*_. Low values of *E*_*h*_ enable agents to obtain more energy within the same time interval and procreate faster. This leads to oscillations in the population size, as the population rapidly increases beyond what the environment can support. As *E*_*h*_ decreases, the amplitude of these oscillations increases, eventually destabilising the system and causing extinction. In the following we consider a parameter change in the value of *E*_*h*_ as a pressure to the system and we consider the resilience of the system against this pressure, and how this resilience is affected by adaptation.

To examine the long-term model behaviour for various values of *E*_*h*_, we approximate time-averaged pdfs based on a single run of both versions of the ABM. The results for a few selected values of *E*_*h*_ are shown in Fig 8. For *E*_*h*_ = 0.1, both pdfs are bimodal, with the peaks corresponding to the extrema of the oscillations (Fig 8a). The distance between the peaks is smaller in the adaptive ABM than in the ABM without adaptation. For *E*_*h*_ = 0.05, both ABMs converge to extinction, as indicated by the peaks at *n* = 0 (Fig 8c). For *E*_*h*_ = 0.07, extinction has occurred only in the ABM without adaptation. The adaptive ABM still has a positive population at the end of the run (Fig 8b).

**Fig 9:**
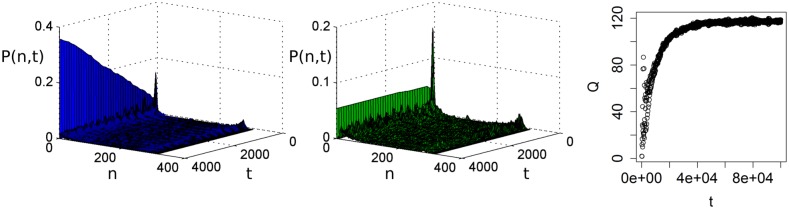
Time-dependent histograms at *E*_*h*_ = 0.07. (a): Histogram of the ABM without adaptation. (b): Histogram of the ABM with adaptation. (c): The value of *Q* as a function of time. The value of *Q* increases as without adaptation an increasing number of runs goes to extinction.

We use 1000 replicate runs of both ABMs to approximate the pdfs as a function of time. The resulting histogram of the ABM without adaptation has a peak at *n* = 0 and the height of the peak increases over time (Fig 9a). Thus, the probability that a population goes extinct remains positive over time, resulting in an increasing number of extinct runs as time progresses. The ABM with adaptation also has a peak at *n* = 0, but the height of this peak is smaller and does not increase after the first few-hundred time-steps (Fig 9b). Thus, initially there is a positive probability of going extinct, but this probability decreases to zero over time. After some time, no more runs go to extinction. The adaptation measure *Q* increases, since the number of runs resulting in extinction increases in the ABM without adaptation (Fig 9c). Around *t* = 50,000, all the model runs of the ABM without adaptation have gone to extinction, after which the adaptation measure no longer varies.

**Fig 10:**
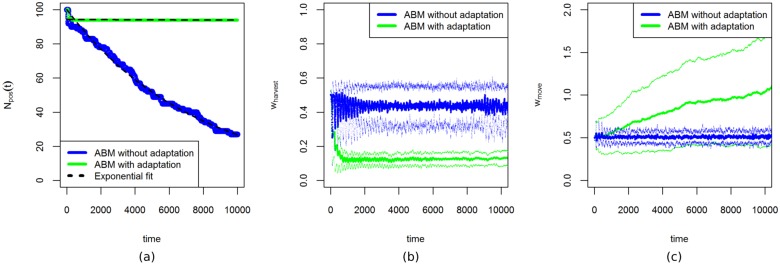
Effect of adaptation on resilience. (a): Plot of the percentage of model runs with a positive population *N*_*pos*_(*t*) as a function of time. We used 100 replicate runs at *E*_*h*_ = 0.07, for both versions of the ABM. The dashed lines correspond to exponential fits (Eq 9). Without adaptation, an increasing number of runs go to extinction, whereas with adaptation there is extinction only on short time-scales. (b): Mean value of *w*_*harvest*_ over the agents of all the 100 replicate runs of the ABMs with adaptation and without adaptation. The upper and lower lines show the mean plus or minus one standard deviation. (c): The mean value of *w*_*move*_ over the agents of all the 100 replicate runs of the ABMs with and without adaptation. The upper and lower lines show the mean plus or minus one standard deviation.

The variation between replicates in the time until the model goes to extinction is caused by stochasticity. To estimate the spread of this timing, we perform 100 model runs for each value of *E*_*h*_ and record the value of *t* where extinction occurs. For *E*_*h*_ = 0.07, the number of runs of the ABM without adaptation with a positive population decreases approximately exponentially over time, whereas for the adaptive ABM extinction occurs only in the first few-hundred time-steps (Fig 10a). If the population does not go extinct during this initial period, then the population will adapt to harvest more often (Fig 10b), and move less often (Fig 10c). These adaptations ensure the long-term survival of the population.

To quantify the timing of the tipping point, we fit an exponential decay function to the number of model runs with a positive population at time *t*,

$$N_{pos}\left(t\right)=N_{pos,0}e^{-\lambda t}.$$ (9)

**Fig 11:**
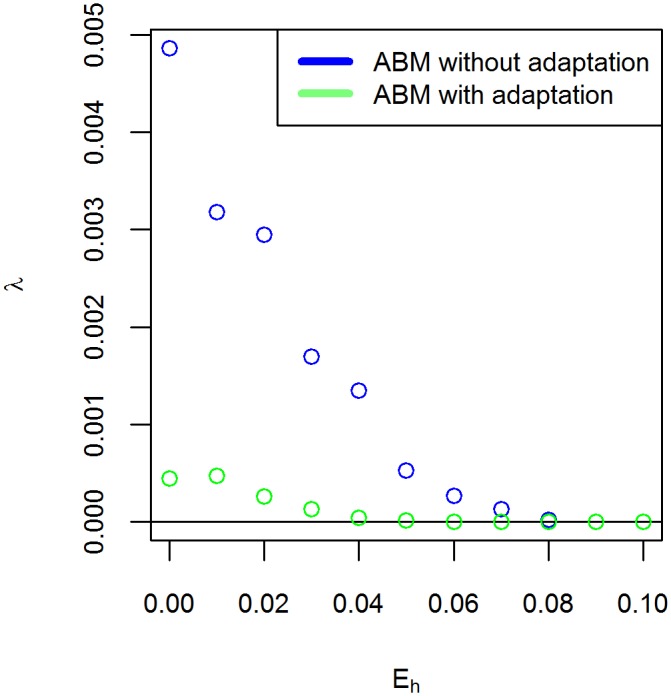
Extinction parameter *λ* as function of *E*_*h*_. The extinction parameter *λ* (Eq 9) as function of the harvest cost *E*_*h*_. The values were estimated by fitting Eq 9 to the output of 100 replicate runs of the ABMs with adaptation and without adaptation. The value of *E*_*h*_ where *λ* reaches zero corresponds to the tipping point where there is no extinction on long time-scales.

Here *N*_*pos*_(*t*) is the number of model runs with a positive population at time *t*, *N*_*pos*,0_ is the total number of model runs and *λ* represents the rate of extinction. High values of *λ* indicate that model runs rapidly go extinct, whereas *λ* = 0 indicates that extinction does not occur at all. We estimate *λ* by fitting Eq 9 to the simulation output using ordinary least squares. The values of the mean squared error of the fits show that Eq 9 gives a good approximation of the simulation output (Table 2). Fig 11 shows that *λ* decreases as *E*_*h*_ increases. Thus, extinction occurs faster for lower values of *E*_*h*_. For all values of *E*_*h*_, the fitted value of *λ* is higher in the ABM without adaptation compared to the ABM with adaptation, indicating that adaptation slows down extinction. We consistently observe that the tipping point where *λ* goes to zero occurs at a lower value of *E*_*h*_ for the ABM with adaptation than for the ABM without adaptation. For example, at *E*_*h*_ = 0.07, the ABM without adaptation has a positive value of *λ*, indicating that all model runs eventually go extinct. On the other hand, in the ABM with adaptation populations survive on long time-scales. Our results thus show that the capacity of the agent population to adapt to its surroundings increases its resilience to circumstances that put pressure on the population.

**Table 2:** Values of the mean squared error (MSE) of the exponential fits to the number of replicate runs with surviving populations (Eq 9).

	*E*_*h*_	0.0	0.01	0.02	0.03	0.04	0.05	0.06	0.07	0.08	0.09	0.10
MSE	ABM without adaptation	2.2	2.0	2.9	2.4	4.9	3.2	3.2	1.9	1.3	4.6	3.5
	ABM with adaptation	5.7	3.3	1.6	1.8	2.1	5.8	5.2	9.5	3.2	7.5	3.5

### Increasing pressure over time

So far, we have considered the effect of adaptation on the ability of the population to cope with a pressure that is constant over time. In the following we will consider a pressure that increases over time, in the form of a gradual decrease in the diffusion-coefficient *D*. For high values of *D* the resource spreads quickly from high density areas to low density areas, whereas for low values this spread is slow. Decreasing values of *D* put pressure on the agent population, because agents need to search more actively for resource. Thus, we introduce a change in the value of *D* over time,

$$\rho =-\frac{dD}{dt}.$$ (10)

**Fig 12:**
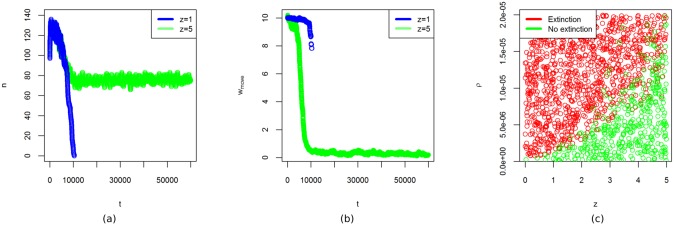
Increasing pressure over time. (a): Number of agents *n* as function of time for a rate of adaptation *z* = 1 (blue) and *z* = 5 (green). In both plots *ρ* = 5 × 10^−5^ Eq (10). (b): Average value of *w*_*move*_ across agents, for the same model runs as in (a). (c): Each point in the figure corresponds to a run of 60,000 time-steps. Red points indicate that the population has gone extinct, whereas for the green points the population has remained positive until the end of the simulation. Extinction occurs if the rate of adaptation *z* is insufficient to cope with the pressure increase *ρ*.

For positive values of *ρ*, the diffusion of the resource gradually slows down during a simulation run, putting pressure on the population. The value of *ρ* determines the rate at which the pressure increases. We expect that if the pressure increases at a high rate, then adaptation must also occur at a high rate for the system to remain resilient. In the model, this rate of adaptation is determined by the random deviation of the agent characteristics upon reproduction. If these deviations are small, then offspring will be very similar to their parents and adaptation will be slow. If the deviations are larger, the differences between offspring and their parents are larger, which enables faster adaptation. The model parameter *z* controls the size of these deviations. We examine the interaction between the rate of pressure increase and the rate of adaptation by running the model for different combinations of *ρ* and *z*. Each run is initiated with parameter values of *D* = 0.1, the energy cost of moving *E*_*m*_ = 0.1 and *w*_*harvest*_ = *w*_*move*_ = 10 for all agents. All other parameters are at the nominal setting. During each run, the pressure is increased by lowering the value of *D* from *D* = 0.1 to until *D* = 0.05. Lower values of *D* lead to extinction regardless of adaptations in the population. If adaptation is slow, then the pressure that results from the decrease of *D* leads to collapse of the population, but if the adaptation is sufficiently fast then a positive population is maintained (Fig 12a). The mean value of *w*_*move*_ across this population shows that agents adapt to move more frequently in search of resource, but that this adaptation is not fast enough for low values of *z* (Fig 12b). Runs for various values of *z* and *ρ* show that there is a critical transition where the pressure on the population increases too fast for adaptations to keep up (Fig 12c). To better understand the resilience of the system to pressure, it is thus important to weigh the rate at which the population is able to adapt against the rate of changes that put pressure on the system.

## Conclusions & discussion

For ABMs to be a useful tool for the assessment of the resilience of social-ecological systems, suitable methodologies for analysing these ABMs are needed. In this paper, we have proposed a methodology for analysing effects of agent adaptation in ABMs, and showed how this adaptation affects resilience. We have illustrated the use of this methodology by applying it to an ABM of consumers competing for a common-pool resource. The method is based on a comparison between the time-averaged pdfs of an adaptive ABM and version of this ABM for which adaptation has been disabled. We consider the difference between these two pdfs as a measure for the effects of adaptation on the model output. This difference is quantified using the earth-mover’s distance [25] as a measure for the adaptation of the system. This measure differs from previously used sensitivity measures based on pdfs (e.g. [26–28]) in that even when different model runs do not overlap in terms of output the earth-mover’s distance is still able to quantify the sensitivity. Note that sensitivity measures quantify the output change or variation as a function of changes or variations in parameter values. Although Eq 1 measures the ‘sensitivity’ of the output to adaptation, it is not a sensitivity measure in the conventional sense because it does not take into account the size of the parameter change. When considering the sensitivity to a continuous parameter, the derivative of Eq 1 with respect to the parameter could be considered as a sensitivity measure. An advantage of the described method over most sensitivity analysis methods is that we explicitly consider the presence of tipping points, and how these tipping points are affected by adaptation. Existing sensitivity analysis methods do not take into account that the model behaviour may be qualitatively different in different regions of parameter space.

To test the potential of the methodology, we have applied it to a test-case ABM of agents competing for a common-pool resource. For this test-case, the methodology shows that adaptation increases the resilience of the system to pressures. This resilience is defined in terms of the amount of pressure the system can cope with before it jumps to an alternative domain of attraction. In our case, this jump corresponds to the tipping point where the agent population goes extinct. Without adaptation, this tipping points always occurs at certain parameter values. Agent adaptation is added in the form of a process of natural selection. As a result of this adaptation, the location of the tipping point shifts, and the boundaries of the domain of attraction become wider. This positive contribution of adaptation to the resilience of the test-case depends on the rate at which adaptation takes place. The analysis of the ABM shows that for a system to be resilient, the rate of adaptation needs to be sufficiently fast to cope with increasing pressures or changing conditions.

Other authors have discussed ABMs similar to our test-case [32–34] In [32, 33] the focus is mostly on the evolution of behavioural strategies for the agents, which are characterised by a neural net. Both studies find that various strategies evolve, ranging from greedy to cooperative. In our ABM the range of possible strategies is smaller, because the ABM contains only two agents parameters that are affected by natural selection. For any given parameter setting the model seems to converge to a dominant strategy, but it depends on the parameter setting what this strategy is. For some settings it corresponds to ‘greedy’ agents that harvest as often as possible, whereas for other settings it corresponds to agents that harvest less frequently in order to let the resource grow. A possible extension to our model would be to include more agent parameters, possibly involving direct interactions between agents such as in [32]. For the present study, our main aim is to examine how agent behaviour affects the resilience of the system as a whole. In [34] the resilience of different stability domains of an ABM is considered, but here the agent behaviour is completely pre-defined, and the system does not contain adaptation.

In this paper we have considered only adaptation through a process of natural selection. Adaptation through natural selection typically takes place on long time-scales relative to other types of adaptation. Furthermore, adaptation through natural selection is reactive in the sense that it that responds to changes in the environment, but cannot respond to changes that agents might foresee in the future. In many CAS, individual agents have the ability to learn from past experiences and to adjust their behaviour according to expected future developments. These types of adaptation can be expected to operate on much shorter time-scales than natural selection [35]. Future work will include investigating types of adaptation that operate on faster time-scales, or in which agents adapt proactively based on foreseen changes. Such fast adaptations might lead to an increase in the resilience of the system as agents are able to quickly adapt to situations and respond to pressures. Alternatively, however, they might also destabilise the system, for example when agents mispredict trends or overspecialise [36]).

In this paper, we have analysed the effects of adaptation in a simulated CAS. The analysis is based on a comparison between a non-adaptive and an adaptive ABM. In order to apply this methodology to a real-life CAS, one thus needs to develop model versions with and without adaptation. To this end, one needs to assess whether the system is adaptive, and what system components and interactions are relevant to understanding this adaptation. Qualitative tools for this kind of assessment are available (e.g. [37]). The methodology in this paper complements such resilience assessments by quantifying the resilience of the system using ABMs.

Our results imply that for studies that aim to assess or to enhance the resilience of social-ecological systems, it is relevant to consider the ability of the system to adapt. When combined with suitable methodologies for sensitivity analysis, ABMs can be a helpful tool to test hypotheses on which factors may contribute to the resilience of social-ecological systems (e.g., [38–41]).

## Supporting information

S1 Appendix Stationarity and ergodicity tests. (DOCX)

S2 Appendix Computation of the earth mover’s distance. (DOCX)

S3 Appendix Results of stationarity and ergodicity tests. (DOCX)

S1 Table Default parameter setting. (DOCX)
