# Supplementary material for: Resilience through adaptation
Source: PLoS One. 2017 Feb 14;12(2):e0171833. doi: 10.1371/journal.pone.0171833 (PMC5308918; doi:10.1371/journal.pone.0171833)
Supplement: S1 Appendix — (DOCX) [file pone.0171833.s001.docx]

**S1 Appendix: Stationarity and ergodicity tests**

For some ABMs stationarity and ergodicity can be proven analytically, without the use of simulation runs (Izquierdo et al. 2009). For many ABMs, however, such a formal proof is not possible because we do not have an analytical description of the processes that generate the output (Grazzini 2012). We then have to rely instead on statistical tests for stationarity and ergodicity of the output of simulation runs. Such tests cannot formally prove stationarity or ergodicity, but may statistically verify these properties. A number of statistical tests are available for this purpose (Philips and Xiao 1998). Most of these tests are parametric tests that require assumptions on the shape of the output pdf, such as the assumption that the output is normally distributed. For ABMs analysis, however, the complexity of the model often makes it difficult to predict the shape of the output pdf (Grazzini 2012). Furthermore, since an investigation of the stationarity is often one of the first steps in model analysis, we may not have investigated the properties of the output pdf before we test the stationarity. In this paper we therefore use only non-parametric tests, which impose no assumptions on the output pdf. The downside of using parametric tests is that they are relatively conservative, i.e. using these tests it is difficult to reject the null-hypothesis that the series is stationary. In the context of analysing data from ABMs, this limitation may be partially addressed by increasing the amount of data that is recorded from the ABM, or by adapting the used significance level.

To test the stationarity we first divide the time-series into a number of short-term time-windows and compute the average of the model output over each window (Grazzini 2012). By averaging over time-windows we aim to remove short-term fluctuations from the time-series. Removing these fluctuations ensures that the window-means can be considered as independent observations of the stochastic process. The stationarity test then tests for the presence of trends in this series of observations. Two commonly used non-parametric tests are the Runs Test (Wald and Wolfowitz 1940; Grazzini 2012; WMO 2006) and the Mann-Kendall trend test (Mann 1945; Kendall 1975; Bendat and Piersol 2006; Yue et al 2002; WMO 2006). The trend test is relatively powerful for the detection of monotonic trends, whereas the runs test is typically more powerful for the detection of fluctuating trends (Bendat and Piersol 2006). We therefore use both tests to verify and supplement each other. The details of both tests will follow below.

To verify ergodicity of the model output we use the same tests, but applied to different samples (Grazzini 2012). The first sample is composed of a single model run, divided into a number of equal length subseries. The second sample is composed of a number of model runs, with the length of each run equal to the length of a subseries of the first sample. We now use the runs test or trend test to compare the means of the subseries of the first sample to the means of the model runs of the second sample. If the model is ergodic, then the two sets of means come from the same pdf. Thus, with the ergodicity test we test the null-hypothesis that the underlying pdfs are equal.

*Runs Test*

To test stationarity to of a time-series, we apply the Runs test with the null-hypothesis that the series is stationary, i.e. the underlying distribution does not change over time. The Runs test considers for each observation in the time-series whether the observation is smaller than, or larger than the mean $E(n_{t})$ over the entire time-series. Based on this comparison, we compute the vector $H_{t}$ so that

$H_{t}= \left\{ \begin{aligned} 0 ;n{}_{t}<E(n_{t}) \\ 1 ;n_{t}>E(n_{t}) \end{aligned} \right.$

where we ignore observations that are equal to the mean. If the output is stationary we expect the deviations from the mean to be randomly distributed over time. If, however, there is a trend then the deviations are not randomly distributed. For example, for an upwards trend $H_{t}$ will have more 0’s for smaller $t$ and more 1’s for larger $t$. Such irregularities in $H_{t}$ are expressed in terms of the number of “runs” $R$. A run is defined as a consecutive series of identical symbols in the vector $H_{t}$. For example, the series $\{0,1,0,0,0,1,1,0\}$ contains 5 runs $(\{0\},\{1\},\{0,0,0\},\{1,1\},\{0\})$. If the time-series is stationary, then the expectation value and the variance of the random variable $R$ are known (Wald and Wolfowitz 1940). For the mean we have

$E\left( R \right)=\frac{2N_{+}N_{-}}{N_{+}+N_{-}}+1$

with $N_{+}$ the number of observations above the total mean and $N_{-}$ the number of observations below the total mean. The variance is

$V\left( R \right)=\frac{2N_{+}N_{-}\left( 2N_{+}N_{-}-N_{+}-N_{-} \right)}{\left( N_{+}+N_{-} \right)^{2}(N_{+}+N_{-}-1)}$

We can thus test for non-stationarity by comparing the value of $R$ measured from a time-series to these equations, which give our null-hypothesis. If the number of runs differs significantly then we reject the null hypothesis that the output is stationary.

The value of $R$ can be affected by dependency between observations that are close in time. For example, if an observation at time $t$ lies above the mean, then the observation at time $t+1$ may also tend to lie above the mean. The resulting number of runs would then be smaller than would be expected based on the above equations. The dependency between observations is removed by averaging the observations over time-windows. A single observation then corresponds to the mean of a window and the vector $H_{t}$ is computed over these window means.

*Trend Test*

The trend test is a commonly used alternative to the runs test for establishing stationarity of a stochastic process (Bendat and Piersol 2006; WMO 2006). The trend test is more powerful for finding monotonic (upwards or downwards) trends, whereas the runs test is better at finding fluctuating trends (Bendat and Piersol 2006). We will thus use both tests to verify and complement each other. As in the runs test, the null-hypothesis is that the underlying distribution of a time-series is stationary. The trend test is based on the value of,

$W=\sum_{t=1}^{N_{m}-1} \sum_{t'=t+1}^{N_{m}} sgn\left( n_{t}-n_{t'} \right)$

where $T$ is the number of observations and

$sgn\left( n \right)= \left\{ \begin{aligned} -1 ;n<0 \\ 0 ; n=0 \\ 1 ;n>0 \end{aligned} \right.$

For a sufficiently long time-series, $W$ is normally distributed with mean and variance,

$E\left( W \right)=0$

$V\left( W \right)=\frac{1}{18}N_{m}\left( N_{m}-1 \right)\left( 2N_{m}+5 \right)-\sum_{g} g(g-1)(2g+5)$

Here the summation $\Sigma_{g}$ runs over all groups of observations that have tied output values $n$ and $g$ is the number of measurements in each of these groups. These equations for the mean and variance describe the null-hypothesis that the actual values of $W$ are tested against. Similar to the runs Test, the trend is affected when there is a dependency between observations that are close in time. We may divide the series into time windows and treat the window averages as individual observations to remove such dependencies.

*References*

Bendat JS, Piersol AG. Statistical Principles, in Random Data: Analysis and Measurement Procedures, Fourth Edition. John Wiley & Sons, Inc. 2010.

Grazzini J. Analysis of the emergent properties: stationarity and ergodicity. Journal of Artificial Societies and Social Simulation. 2015:15(2).

Izquierdo LR, Izquierdo SS, Galan JM, Santos JI. Techniques to understand computer simulations: Markov chain analysis. Journal of Artificial Societies and Social Simulation. 2009:12(1).

Kendall MG. Rank Correlation Methods, Griffin, London. 1975.

Mann HB. Nonparametric tests against trend, Econometrica. 1945:13:245–259.

Phillips PC, Xiao Z. A primer on unit root testing. Journal of Economic Surveys. 1998:12(5): 423-470.

Wald A, Wolfowitz J. On a test whether two samples are from the same population. The Annals of Mathematical Statistics. 1940:11(2):147-162.

World Meteorological Organization (WMO). Guide to hydrological practices, volume II, management of water resources and application of hydrological practices. Chapter 5, Extreme value analysis, WMO-No. 168 , 6th Ed., Geneva, 2009.

Yue S, Pilon P, Phinney B, Cavadias G. The influence of autocorrelation on the ability to detect trend in hydrological series. Hydrological Processes. 2002:16(9):1807-1829.
