# Supplementary material for: Resilience through adaptation
Source: PLoS One. 2017 Feb 14;12(2):e0171833. doi: 10.1371/journal.pone.0171833 (PMC5308918; doi:10.1371/journal.pone.0171833)
Supplement: S2 Appendix — (DOCX) [file pone.0171833.s002.docx]

**S2 Appendix: Computation of the earth mover’s distance**

Computing the earth-mover's distance between a pair of distributions amounts to finding the minimal 'work' needed to change on distribution into the other. In this paper, we will deal with pairs of one-dimensional discrete output distributions, $P_{a}(n)$ and $P_{b}(n)$, where we have dropped the time-dependecy for convenience. The earth-mover's distance is then written as (Ling & Okada, 2007),

$$d_{e} = \min\left( \sum_{j,k} g\left( j,k \right)d\left( j,k \right) \right)$$

with the constraints,

$$\sum_{k} g\left( j,k \right)=P_{a}(j) \forall j$$

$$\sum_{j} g(j,k)= P_{b}\left( k \right)\forall k$$

$$g\left( j,k \right)\geq0 \forall j,k$$

with $g(j,k)$ the flow between output values, $d(j,k)$ the distance between output values, and the indices $j$ and $k$ running over all possible output values. The first two constraints ensure that the flow is such that the distribution $P_{a}(j)$ is transformed into $P_{b}(k)$. The third constraint ensures that mass is moved from $P_{a}(j)$ to $P_{b}(k)$, and not the other way around. The first equation ensures that the flow is chosen such to minimise the required 'work' $g(j,k)d(j,k)$. For two pdfs of a single output variable, this minimisation is accomplished by going through all consecutive pairs of output values, and keeping track of the amount of mass that needs to be transported, such as in the following piece of pseudo-code.

$u(0)=0$

$for i=1:N_{b}$

$u\left( i \right)=u\left( i-1 \right)+P_{a}\left( i \right)-P_{b}\left( i \right)$

$end$

$d_{e}=\sum_{i} |u\left( i \right)|$

Here the vector $u\left( i \right)$ stores the amount of mass that needs to be transported between consecutive bins, and $N_{b}$ is the total number of bins.

*References*

Ling H, Okada K. An efficient earth mover's distance algorithm for robust histogram comparison. IEEE transactions on pattern analysis and machine intelligence. 2007;29(5):840--853.

Rubner Y, Tomasi C, Guibas LJ. A metric for distributions with applications to image databases. In: Computer Vision, 1998. Sixth International Conference on. IEEE; 1998. p. 59--66.
