# Supplementary material for: Resilience through adaptation
Source: PLoS One. 2017 Feb 14;12(2):e0171833. doi: 10.1371/journal.pone.0171833 (PMC5308918; doi:10.1371/journal.pone.0171833)
Supplement: S3 Appendix — (DOCX) [file pone.0171833.s003.docx]

**S3 Appendix: Results of stationarity and ergodicity tests**

*ABM without adaptation*

We investigate the convergence of the output of the ABM without adaptation to a stationary state in the nominal setting. The mean of the model output $n$ over 10,000 replicates as a function of time (Figure S3 1a) shows that the output initially oscillates, but stabilizes before $t=1000$, after which it shows minor fluctuations around a mean value. The estimated output pdfs at $t=1000$ and $t=2000$ are approximately equal (Figure S3 1b) and the model thus seems to be stationary. We further verify stationarity by performing a runs test (Grazzini 2012) and a trend test (Bendat and Piersol 2006; WMO 2006) between $t=1000$ and $t=2000$ on each of the separate 1000 model runs. We use a 5% significance level. If the model is stationary we thus expect that the null-hypothesis will be rejected for 5% of the model runs. If we treat each time-step as an independent observation, then the null-hypothesis is rejected for most model runs. These rejections occur because the observations at times $t$ and $t-1$ are not independent, but related through the processes of birth and death. This dependence is revealed by plots of the autocorrelation function (ACF) and the partial autocorrelation function (PACF). The ACF is the correlation of the time series with itself at different lags in time. Figure S3 2a shows that the ACF decreases only slowly as the lag is increased. Even at a lag of 100 time-steps, there is a significant autocorrelation. The PACF is the correlation of the time series with itself at different lags in time, that is not explained by correlations at smaller lags. The PACF shows a strong peak at a lag of 1 time-step, but is not significant at a lag of two time-steps (Figure S3 2b). From this finding we can conclude that the correlation between larger lags is caused by the correlation at lag 1. To test the stationarity, we remove the dependence between observations by dividing the series into a number of equal length windows. If the windows are sufficiently long, then the window means of the output can be considered as independent observations of the output. The rejection rate then converges to around 5%, indicating that the model is stationary (Figure S3 3). The trend test requires a shorter window length for this convergence than the runs test. This is consistent with the finding of Bendat and Piersol (2006) that the trend test tends to be less sensitive to fluctuating trends than the runs test. We conclude from Figures S3 1 and S3 3 that the model output is stationary between $t=1000$ and $t=2000$.

| \| 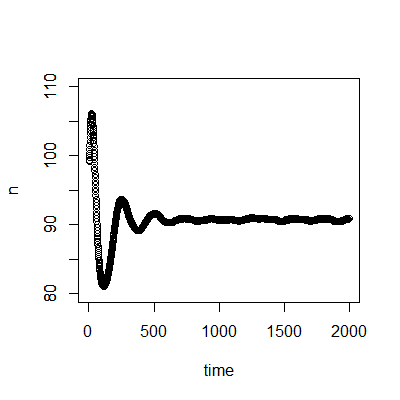 \| 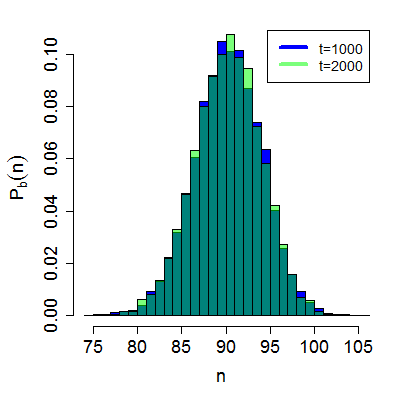 \|  \| \| --- \| --- \| --- \|   (a) (b)  S3 Figure 1(a): The model output $\boldsymbol{n}$ of the ABM without adaptation, averaged over 1000 replicate runs in the nominal parameter setting as function of time. (b): Histograms of the ABM without adaptation at $\boldsymbol{t=1000}$ (blue) and at $\boldsymbol{t=2000}$ (green).  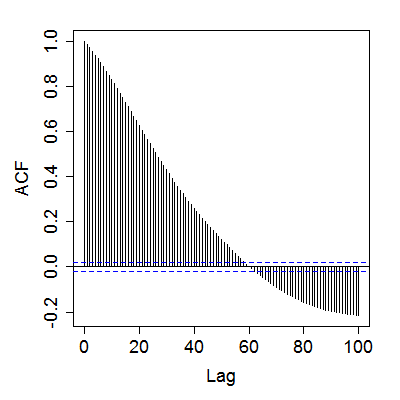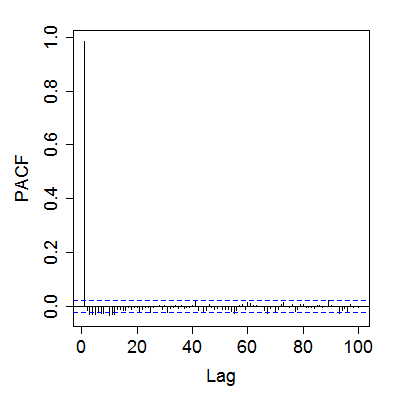  **S3 Figure 2(a): Plot of the ACF as function of the lag. The dashed blue lines show the 5% confidence interval. (b): Plot of the PACF as function of the lag. The dashed blue lines show the 5% confidence interval.** |  |  |
| --- | --- | --- | --- | --- | --- |


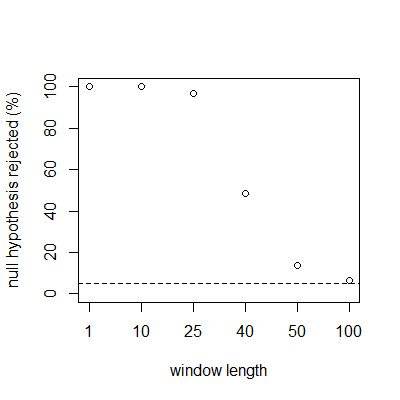

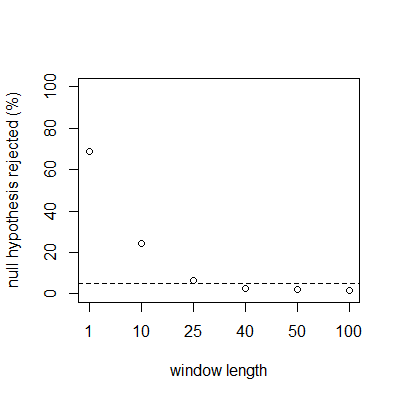


1. (b)

Figure S3 3(a): Percentage of replicate runs for which the runs test rejects the null-hypothesis of stationarity for the ABM without adaptation. On the horizontal axis is the window length, i.e. the number of time-steps over which the output is averaged. The dashed line indicates the 5% significance level. (b): Same as (a), but using the trend test instead of the runs test.

For the ergodicity test we compare the output of a single run measured over a long time to the output of a large number of replicate runs, measured at a set time. The pdf of a single model run of 100,000 time-steps is approximately equal to the pdf of 10,000 model runs at $t=1000$ (Figure S3 4a). This indicates that the model is ergodic. To further verify ergodicity we follow the procedure of Grazzini (2012). We divide the long model run into 100 subseries of 1000 time-steps. The means of these subseries compose the first sample of the ergodicity test. The second sample consists of 1000 model runs, each averaged between $t=1000$ and $t=2000$. A visual comparison between the two samples is shown in Figure S3 4b. A runs test does not reject the null-hypothesis that the two samples come from the same distribution. Based on this test and the visual comparison of the pdfs, we conclude that the model is ergodic.


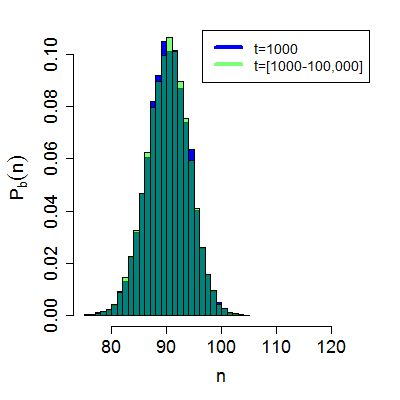

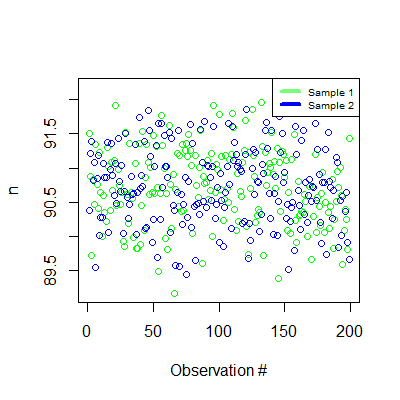


1. (b)

Figure S3 4(a): Histograms of the model output of the ABM without adaptation. The blue histogram is measured over 10,000 replicate runs at $\boldsymbol{t=1000}$, and the green histogram over the time-steps of a single model run between $\boldsymbol{t=1000}$ and $\boldsymbol{t=100,000}$. (b): Samples used for the runs test for ergodicity of the ABM without adaptation. The green sample points are the averaged output of a single model run over a window of 50 time-steps. The blue sample points each correspond to a different model run, averaged between $\boldsymbol{t=1000}$ and $\boldsymbol{t=2000}$.

*Adaptive ABM*

For the adaptive ABM, the results of the stationarity test are similar to the case without adaptation. The model output stabilises before $t=1000$ and does not seem to significantly change between $t=1000$ and $t=2000$ (Figure S3 5). A runs test and trend test for stationarity confirm that the output is stationary, as long as the window length is sufficiently long to remove short term fluctuations from the output (Figure S3 6). The model thus appears to be stationary between $t=1000$ and $t=2000$.

| 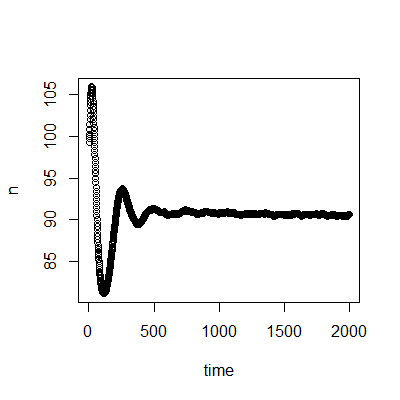 | 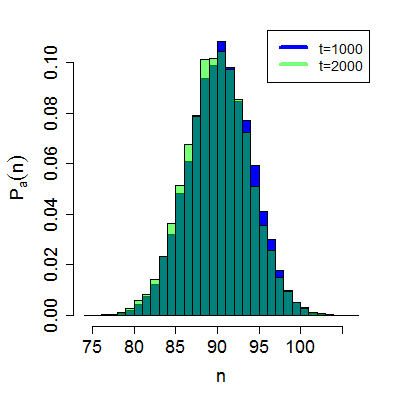 |
| --- | --- |

1. (b)

Figure S3 5(a): The model output $\boldsymbol{n}$ of the ABM with adaptation, averaged over 1000 replicate runs in the nominal parameter setting as function of time. (b): Histograms of the ABM with adaptation at $\boldsymbol{t=1000}$ (blue) and at $\boldsymbol{t=2000}$ (green).


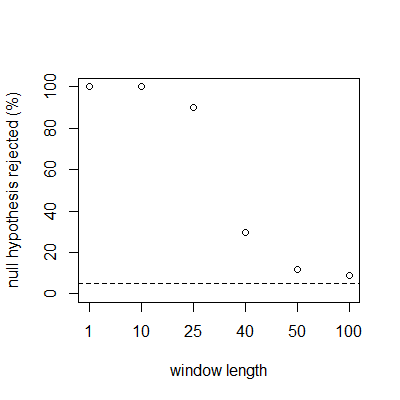

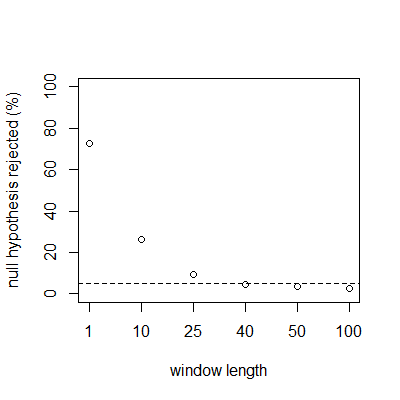


(a) (b)

Figure S3 6(a): Percentage of replicate runs for which the runs test rejects the null-hypothesis of stationarity for the ABM without adaptation. On the horizontal axis is the window length, i.e. the number of time-steps over which the output is averaged. The dashed line indicates the 5% significance level. (b): Same as (a), but using the trend test instead of the runs test.

We use an ergodicity test to explore the long-term model behaviour. The adaptive ABM is not ergodic. The pdf of a long model run measured over time clearly differs from the pdf at $t=1000$ (Figure S3 7). Over time, the population gradually adapts, which leads to an increase in the population size. The trend test shows that there is a positive trend in the adaptive ABM. Based on the series of window means in Figure S3 7b, we used the Theil Sen method to estimate the slope. This yields a slope of 1.20 * 10^-4^ agents per time-step.


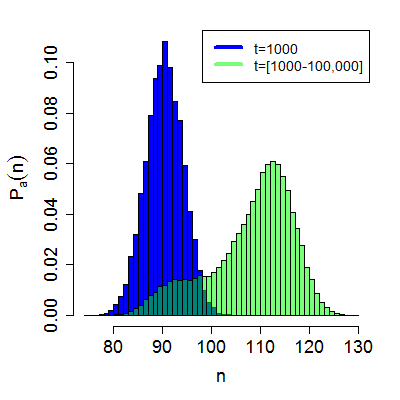

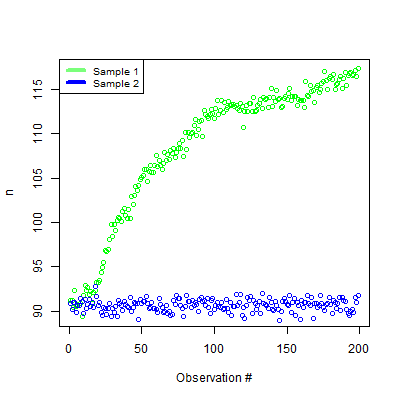


Figure S3 7(a): Histograms of the model output of the ABM with adaptation. The blue histogram is measured over 10,000 replicate runs at $\boldsymbol{t=1000}$, and the green histogram over the time-steps of a single model run between $\boldsymbol{t=1000}$ and $\boldsymbol{t=100,000}$. (b): Samples used for the runs test for ergodicity of the ABM with adaptation. The green sample points are the averaged output of a single model run over a window of 50 time-steps. The green sample points each correspond to a different model run, averaged between $\boldsymbol{t=1000}$ and $\boldsymbol{t=2000}$.

*References*

Bendat JS, Piersol AG. Statistical Principles, in Random Data: Analysis and Measurement Procedures, Fourth Edition. John Wiley & Sons, Inc. 2010.

Grazzini J. Analysis of the emergent properties: stationarity and ergodicity. Journal of Artificial Societies and Social Simulation. 2015:15(2).

World Meteorological Organization (WMO). Guide to hydrological practices, volume II, management of water resources and application of hydrological practices. Chapter 5, Extreme value analysis, WMO-No. 168 , 6th Ed., Geneva, 2009.
