# Supplementary material for: Resilience through adaptation
Source: PLoS One. 2017 Feb 14;12(2):e0171833. doi: 10.1371/journal.pone.0171833 (PMC5308918; doi:10.1371/journal.pone.0171833)
Supplement: S1 Table — (DOCX) [file pone.0171833.s004.docx]

**S4 Table**

Table: Default setting of model parameters

| Symbol | Description | Dimensions/range | Default value |
| --- | --- | --- | --- |
| *c* | Efficiency | J kg^-1^ | 0.9 |
| *D* | Diffusion coefficient | km^2^day^-1^ | 0.1 |
| *E_b_* | Birth energy | J | 5 |
| *E_h_* | Harvest cost | J | 0.3 |
| *E_m_* | Cost of energy maintenance | J | 0.1 |
| *E_move_* | Move cost | J | 0.5 |
| *K* | Carrying capacity | kg | 2 |
| *n*0 | Initial number of agents | 0,1,2, ... | 100 |
| *r* | Growth rate | s^-1^ | 0.1 |
| *R*_0_ | Initial resource | 0-1 (proportion of *K*) | 1 |
| *R_max_* | Maximum harvest | kg | 0.5 |
| *R_unc_* | Uncertainty of resource estimations | kg | 0.1 |
| *v_b_* | Birth coefficient | J^-1^ | 10 |
| *v_d_* | Mortality coefficient | J^-1^ | 10 |
| *z* | Variation in offspring traits | [0,→> | 0.2 |
